# Development of eosinophilic pneumonia from eosinophilic bronchiolitis without asthma: A case report

**DOI:** 10.1016/j.rmcr.2023.101901

**Published:** 2023-07-23

**Authors:** Erika Mikami, Kenichiro Atsumi, Hirokazu Iso, Takahiro Suzuki, Satoru Matsuki, Kakeru Hisakane, Koji Nagata, Masahiro Seike, Takashi Hirose

**Affiliations:** a Department of Pulmonary Medicine and Oncology, Nippon Medical School Tama Nagayama Hospital, Tokyo, Japan; b Department of Pathology, Nippon Medical School Tama Nagayama Hospital, Tokyo, Japan; c Department of Pulmonary Medicine and Oncology, Graduate School of Medicine, Nippon Medical School, Tokyo, Japan

**Keywords:** Eosinophilic bronchiolitis, Bronchial asthma, Eosinophilic pneumonia, Hypereosinophilic obliterative bronchiolitis

## Abstract

Eosinophilic bronchiolitis is a disease concept reported in Japan in 2001, that presents with bronchiolitis accompanied by eosinophilia in the blood and lungs. In 2013, hypereosinophilic obliterative bronchiolitis, as a group of disease presenting with eosinophilic bronchiolitis, was proposed in France. The relationship between eosinophilic bronchiolitis and other eosinophil-related diseases has not been clarified. Herein, we report the case of a 56-year-old female patient with eosinophilic bronchiolitis without asthma, which developed into eosinophilic pneumonia. Treatment with oral prednisone improved the respiratory function. According to the clinicopathological findings in this case, eosinophilic bronchiolitis may be a different disease from asthma.

## 1 Introduction

Eosinophilic bronchiolitis is a disease concept first proposed in Japan in 2001. It is a disease characterized by eosinophilia in blood and the bronchoalveolar lavage fluid (BALF), as well as pathological and radiological manifestation of bronchiolitis [1]. Thus far, few cases of eosinophilic bronchiolitis have been reported. In 2013, hypereosinophilic obliterative bronchiolitis (HOB) was proposed in France; the definition of this disease was based on the following criteria: 1) blood eosinophil cell count >1 G L (-1) and/or BALF eosinophil count >25%; 2) persistent airflow obstruction despite treatment with high-dose inhaled bronchodilators and corticosteroids; and 3) eosinophilic bronchiolitis at lung biopsy and/or direct signs of bronchiolitis (e.g., centrilobular nodules and branching opacities) on computed tomography (CT) [2]. It was suggested that HOB is a group of diseases that present with eosinophilic bronchiolitis, including secondary forms, and may be characterized by a similar pathogenesis to that of eosinophilic asthma. Moreover, recognizing HOB and distinguishing it from asthma is important because the use of oral corticosteroid (OCS) therapy may result in marked improvement. In this article, we report the case of eosinophilic bronchiolitis without bronchial asthma, which developed into eosinophilic pneumonia. The relationship between eosinophilic bronchiolitis and other eosinophil-related diseases, including bronchial asthma, has not been clarified. Further investigation and case reports are needed before the term “eosinophilic bronchiolitis” can be widely recognized as an independent disease entity.

## 2 Case report

A 56-year-old Japanese female with no history of smoking visited our hospital in May 2022 with dyspnea on exertion and severe wet cough. She received treatment with a high-dose inhaled corticosteroid plus a long-acting β2-agonist and a leukotriene receptor antagonist for suspected bronchial asthma in November 2021. Treatment with clarithromycin was initiated in March 2022; however, the symptoms did not improve. The patient had a history of Graves’ disease, which was in remission at that the time of treatment. Her family history was not relevant to the present disorder. Her occupation is clerical, and she does not keep pets.

**Fig. 1:**
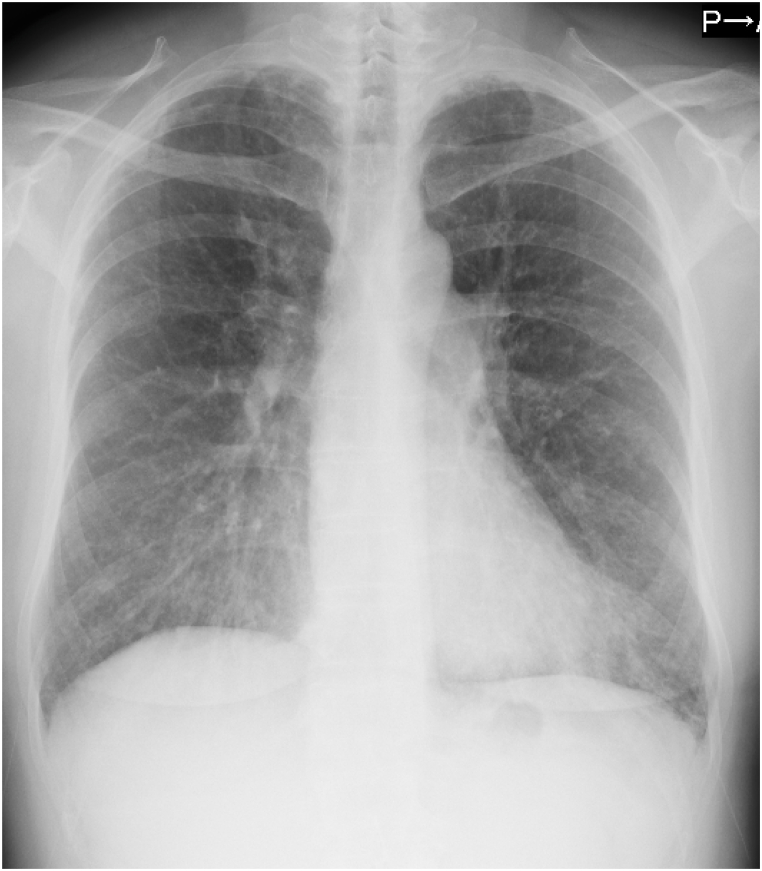
Chest X-ray showing diffuse granular shadows in both lungs.

**Fig. 2:**
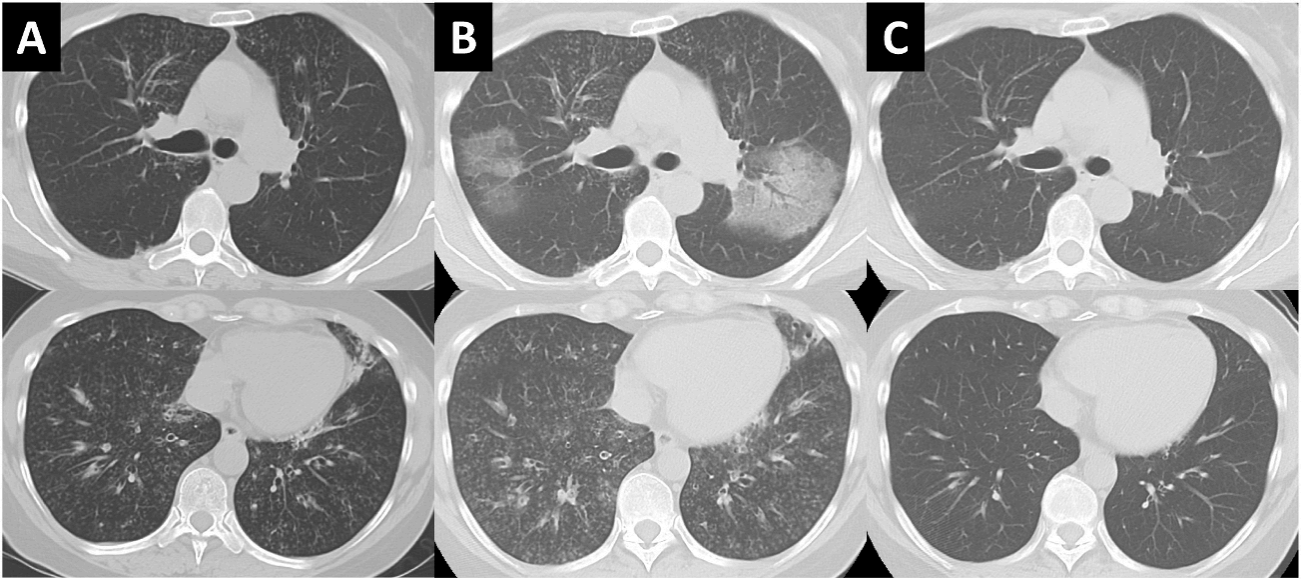
(A) Chest computed tomography (CT) scan at first examination showing diffuse centrilobular nodules and bronchial wall thickening. (B) Pre-treatment chest CT scan after bronchoscopy showing a diffuse nodular shadow and new ground-grass opacity in the right middle and left lower lobes. (C) Two months after the initiation of treatment, chest CT scan showed that both the ground-grass opacity and nodular shadow had disappeared.

On admission, her oxygen saturation was 93% on room air, and there was no wheezing in either of the lung fields. Blood testing revealed eosinophilia (3,010 μ/L) and elevation of the fractional exhaled nitric oxide (FeNO) levels [80ppb]. The levels of total IgE were not elevated (120 IU/mL), and testing for the presence of specific IgE against Aspergillus yielded negative results. Similarly, tests for myeloperoxidase- and proteinase 3-*anti*-neutrophil cytoplasmic antibodies were negative (Table 1). Chest X-ray and high-resolution computed tomography (HRCT) showed diffuse centrilobular granular shadows and bronchial wall thickening (Fig. 1, Fig. 2A). Regarding pulmonary function, forced expiratory volume in 1 s was 1.29 L (51% predicted), forced vital capacity was 2.10 L (67.1% predicted), and diffusing capacity for carbon monoxide was 44.2%. Bronchodilator reversibility testing with salbutamol yielded negative findings. Nasopharyngeal fiber examination did not reveal swelling or thickening of the nasal mucosa, which can be observed in sinusitis. Moreover, head CT did not show evidence suggestive of sinusitis.

**Table 1:** Blood biochemical examination on admission.

Hematology			Infection		
WBC	10,100	/μL	T-SPOT	(－)	
Neutro	46.0	%	MAC antibody	<0.5	U/mL
Lymph	20.9	%	HIV	(－)	
Eosino	29.8	%	human T-cell leukemia virus type-1 antibody	(－)	
Mono	3.1	%			
Baso	0.2	%	β-D-glucan	17.4	pg/mL
RBC	498	× 10^4^/μL	*Aspergillus* antigen	(－)	
Hb	15.1	g/dL	Mycoplasma	<40	×
Plt	34.2	× 10^4^/μL	pneumoniae antibody		

Biochemistry			Serology		
Alb	4.2	g/dL	RF	62	IU/mL
AST	19	U/L	Antinuclear antibody	<40	×
ALT	13	U/L	IgG4	229	mg/dL
LDH	314	U/L	IgA	161	mg/dL
CK	139	U/L	IgE	120	mg/dL
BUN	12.1	mg/dL	Anti CCP antibody	0.5	U/mL
Cre	0.72	mg/dL	MPO-ANCA	(－)	IU/mL
Na	141	mmol/L	PR3-ANCA	(－)	IU/mL
K	4.2	mmol/L	Anti Trichosporon asahii antibody	(－)	U/mL
CRP	2.04	mg/dL			
KL-6	317	U/mL	Anti SS-A antibody	(－)	
HbA1c	6.4	%	TARC	891	pg/mL
ACE	12.1	U/L

**Fig. 3:**
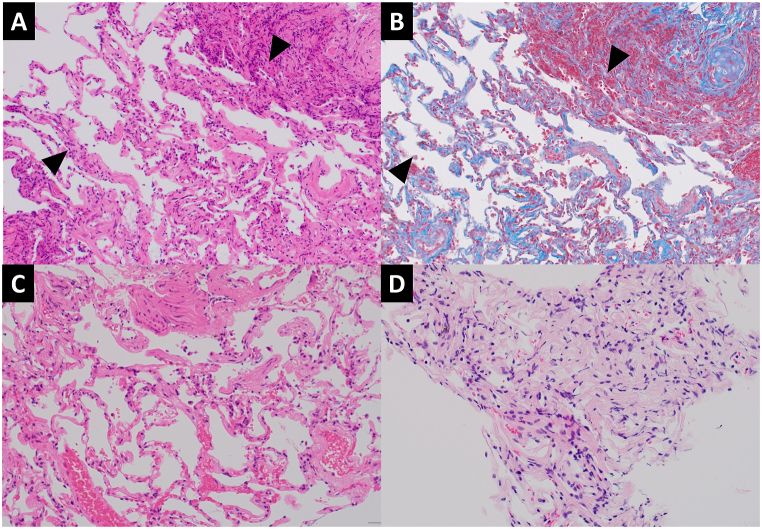
(A) Hematoxylin-and-eosin (H&E) staining showing inflammatory cell infiltration (mainly eosinophilic) in the bronchiolar region (arrow). (B) Masson trichrome staining showing reddish eosinophils, mainly in the bronchiolar region (arrow). (C) Pathology of the alveolar showing slight eosinophilic infiltration. (D) Pathology of the main bronchus showing exudation of chronic inflammatory cells without eosinophils.

**Fig. 4:**
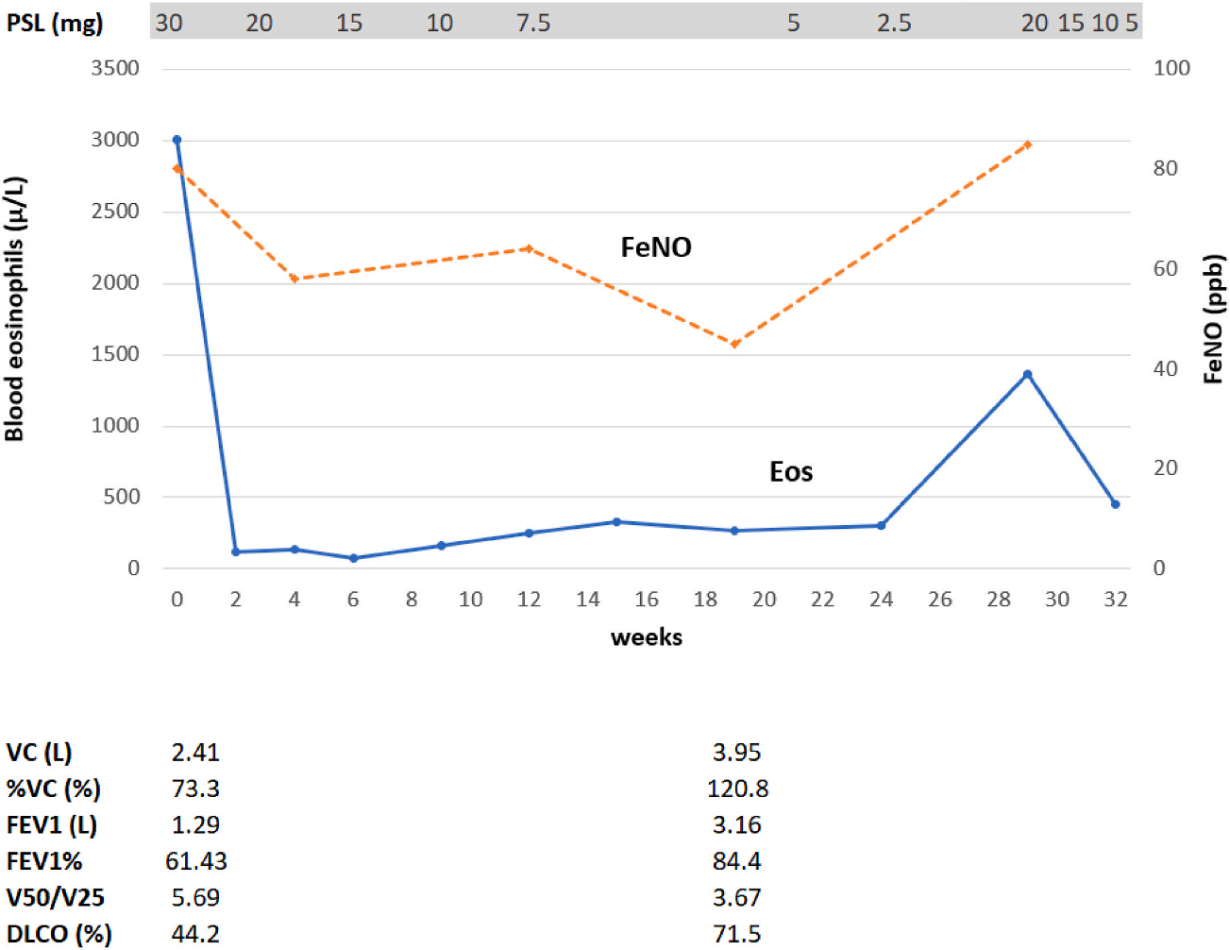
Clinical course. Following therapy with an oral corticosteroid, the peripheral blood eosinophil count and levels of fractional exhaled nitric oxide (FeNO) were decreased, and the respiratory function was improved. However, these values were elevated when the dose of oral prednisone was reduced to 2.5 mg.

Bronchoscopy was performed in the hospital. BAL was performed in the right middle lobe and the total recovery rate of the BALF was 55% (72/130 ml). A transbronchial lung biopsy was randomly performed from the right upper, middle, and lower lobes; in addition, specimens were also collected from the right second carina. Chest X-ray the day after the bronchoscopy showed no new shadows. Specimens randomly collected from peripheral pulmonary lesions showed infiltration of inflammatory cells, mainly eosinophils, into the bronchiolar and alveolar regions. There were markedly fewer eosinophils in the alveolar region than in eosinophilic pneumonia; eosinophils were mainly concentrated in the bronchiolar region (Fig. 3A–C). In contrast, marked infiltration of eosinophils was not observed in specimens collected from the second carina, which showed exudation of chronic inflammatory cells (Fig. 3D). Unlike neutrophils (2%), eosinophils were elevated in BALF (13%). Based on bronchoscopy and blood test findings, the differential disease showing diffuse centrilobular granular shadows such as diffuse panbronchiolitis (DPB), human T-cell lymphotropic virus type-1 associated bronchiolo-alveolar disorder, autoimmune disease including IgG4-related disease and allergic bronchopulmonary aspergillosis were negative. We diagnosed eosinophilic bronchiolitis. Two weeks after bronchoscopy, chest X-ray examination revealed worsening of the shadows. HRCT showed new ground-glass opacity in both lungs. Because lung biopsy was performed on the right lung only, we considered the newly appeared shadows to indicate that this case had progressed to eosinophilic pneumonia (Fig. 2B). Once-daily oral treatment with 30 mg of prednisone was initiated. After treatment, the respiratory function was markedly improved (Fig. 4), and HRCT showed that the shadows had disappeared (Fig. 2C). The dose of prednisone was reduced by 5–10 mg every 2 weeks, with continuous monitoring of the imaging and blood test data. However, when the dose of prednisone dose was reduced to 2.5 mg, the count of peripheral blood eosinophils and FeNO levels were elevated. Therefore, the dose of prednisone was increased for a short period of four weeks. Currently, the patient receives maintenance therapy with 5 mg of prednisone.

## 3 Discussion

Based on the present case, there is a high probability that eosinophilic bronchiolitis and bronchial asthma are distinct diseases. To our knowledge, this is the first report of the development of eosinophilic pneumonia from eosinophilic bronchiolitis.

Thus far, all cases of eosinophilic bronchiolitis have been reported in Japan. An association of eosinophilic bronchiolitis with other eosinophilic diseases, including bronchial asthma, is often suggested [1,3,[5], [6], [7]]. However, a definitive conclusion regarding this potential association has not been reached. Notably, eight of the 10 cases reported thus far were complicated by bronchial asthma (Table 2). The high rate of complications suggests that eosinophilic bronchiolitis may be an extension of bronchial asthma. However, diurnal variation and wheezing were not detected in the present case. In addition, treatment with an inhaled corticosteroid plus a long-acting β2-agonist was ineffective, and the results of bronchodilator reversibility testing were negative. In general, HRCT findings in bronchial asthma include bronchial wall thickening, bronchiectasis and bronchiolar occlusion by mucus [11]. Although structural changes in the small airways such as centrilobular prominence and air trapping may be seen in severe bronchial asthma, small centrilobular opacities are reported to be seen less frequently. Based on the above, this patient was diagnosed with eosinophilic bronchiolitis without bronchial asthma. The pathological findings also suggested that eosinophilic bronchiolitis is a distinct disease from bronchial asthma. Pathological findings of eosinophilic bronchiolitis reported in the past include massive eosinophilic infiltration in the bronchiolar walls, mucus plugs within the bronchiolar lumina and subepithelial fibrosis in the distal airway mucosa [1,5,6,9,10]. In the present case, there was no atypia in the epithelium and no mucus plug. However, eosinophilic infiltrate in the fiber connective tissue of the bronchiolar region was evident. In addition to, there was little eosinophilic infiltration in the specimens collected from the main bronchi. Furthermore, there was no thickening of the basement membrane or hyperplasia of the bronchial smooth muscle, which are characteristic features of bronchial asthma pathology. Consequently, we suggest that eosinophilic bronchiolitis is unlikely to be an extension of bronchial asthma, and should be distinguished from it.

**Table 2:** Clinical characteristics of patients with eosinophilic bronchiolitis reported in previous studies, including our patient.

No.	Author	Year	Age/Sex	Peripheral blood eosinophil count (/μL)	Eosinophil count in BALF (%)	FeNO levels (ppb)	Bronchial asthma	Eosinophilic pneumonia	Sinusitis
1	Takayanagi et al. [1]	2001	46/M	6,840	91	－	－	－	－
2	Fukushima et al. [3]	2010	56/F	2,903	68.7	184.4	＋	－	＋
3	Kobayashi et al. [4]	2015	73/M	1,285	－	－	＋	－	＋
4	Sato et al. [5]	2017	35/M	(12.4%)	24.6	－	＋	－	－
5	Tomyo et al. [6]	2018	60/F	790	28.5	72	＋	＋	－
6	Takeshita et al. [7]	2019	29/M	745	44	78	＋	－	－
7	Takano et al. [8]	2021	83/F	1,580	74	－	＋	＋	＋
8	Sugino et al. [9]	2021	53/F	3,480	49.4	53	＋	－	＋
9	Sasaki et al. [10]	2022	31/M	1,976	67	165	＋	－	－
10	Present case	2023	56/F	3,010	13	80	－	＋	－

In the 10 reported cases of eosinophilic bronchiolitis, the complications of each disease (i.e., eosinophilic pneumonia, bronchial asthma, and sinusitis) did not correlate with the eosinophil count in peripheral blood and BALF, or the levels of FeNO. In addition, there was no correlation with the severity of disease. The complication rate of bronchial asthma was high, whereas those of eosinophilic pneumonia and sinusitis were low. In eosinophilic pneumonia, eosinophil infiltration is generally observed mainly in the alveolar region rather than the bronchioles [12,13]. In the present case, eosinophilic infiltration was concentrated in the bronchiolar region, with only a few eosinophils observed in the alveolar region. In addition, there was no interstitial edema, fibrin deposition, or disruption of the basement membrane observed in eosinophilic pneumonia. These findings suggest that the strong eosinophilic infiltration in the bronchiolar region may have been extended into the alveolar region. The most common CT findings in acute eosinophilic pneumonia is bilateral ground grass opacity [13]. Similar finding is seen in this case, indicating that eosinophilic bronchiolitis may progress to eosinophilic pneumonia.

In this case, inhaled corticosteroid therapy was ineffective. One reason for this is that the inhaled corticosteroid may not have reached the lesions because the bronchiolar lesions in eosinophilic bronchiolitis were more peripheral compared to bronchial asthma. Furthermore, in this case, the eosinophil count in peripheral blood and levels of FeNO rose again when the daily dose of oral prednisone was reduced to 2.5 mg. Among the 10 previously reported cases, therapy with OCS could be discontinued in four cases in which biologic agents were introduced. However, the remaining six cases were being tapered off of oral prednisone or relapsed when the daily dose of oral prednisone was decreased to 5–15 mg. Therefore, patients with disease relapse continued to receive 5–15 mg of oral prednisone daily as a maintenance dose. Regarding HOB, some patients relapsed when the daily dose of prednisone was reduced to 10–20 mg. Therefore, it has been suggested that long-term therapy with OCS is necessary in such cases [2]. Although eosinophilic pneumonia is associated with a high relapse rate, it is possible to wean off therapy with OCS [12,14]. However, in the treatment of eosinophilic bronchiolitis, it may be difficult to discontinue therapy with OCS as in eosinophilic pneumonia. This suggests that eosinophilic bronchiolitis is a chronic airway disease.

Similar to eosinophilic bronchiolitis, DPB is a disease with lesions specifically in the bronchiolar region. DPB is common in East Asia, and its association with the HLA-Bw54 antigen was reported by Sugiyama et al., in 1990 [15]. Eosinophilic bronchiolitis has also been reported mostly in Japan, and its racial dependence and genetic factors should be pursued.

## 4 Conclusion

This case showed that eosinophilic bronchiolitis may progress to eosinophilic pneumonia. Eosinophilic bronchiolitis is linked to a high complication rate of bronchial asthma. However, according to this case, eosinophilic bronchiolitis and bronchial asthma may be distinct diseases.

## Declaration of competing interest

The authors have no potential conflicts of interest to declare.
